# Supplementary material for: Structural basis for recognition of transcriptional terminator structures by ProQ/FinO domain RNA chaperones
Source: Nat Commun. 2022 Nov 18;13:7076. doi: 10.1038/s41467-022-34875-5 (PMC9674577; doi:10.1038/s41467-022-34875-5)
Supplement: Supplementary file 1 — SUPPLEMENTARY FIGURES [file 41467_2022_34875_MOESM1_ESM.pdf]

Structural basis for recognition of transcriptional terminator structures by ProQ/FinO domain RNA chaperones

Hyeong Jin Kim[1], Mazzen Black[1], Ross A. Edwards[1], Flora Peillard-Fiorente[2], Rashmi Panigrahi[1], David Klingler[3], Reiner Eidelpes[3], Ricarda Zeindl[3], Shiyun Peng[1], Jikun Su[1], Ayat R. Omar[1], Andrew M. MacMillan[1], Christoph Kreutz[3], Martin Tollinger[3], Xavier Charpentier[2], Laetitia Attaiech[2,*], and J. N. Mark Glover[1,*]

[1] Department of Biochemistry, University of Alberta, Edmonton, AB, T6G2H7, Canada

[2] CIRI, Centre International de Recherche en Infectiologie, Team "Horizontal gene transfer in bacterial pathogens", Inserm, U1111, Université Claude Bernard Lyon 1, CNRS, UMR5308, Ecole Normale Supérieure de Lyon, Univ Lyon, 69100, Villeurbanne, France

[3] Institute of Organic Chemistry, Center for Molecular Biosciences Innsbruck (CMBI), University of Innsbruck, Innrain 80/82, 6020 Innsbruck, Austria

*To whom correspondence should be addressed: laetitia.attaiech@univ-lyon1.fr, mark.glover@ualberta.ca

SUPPLEMENTARY FIGURES

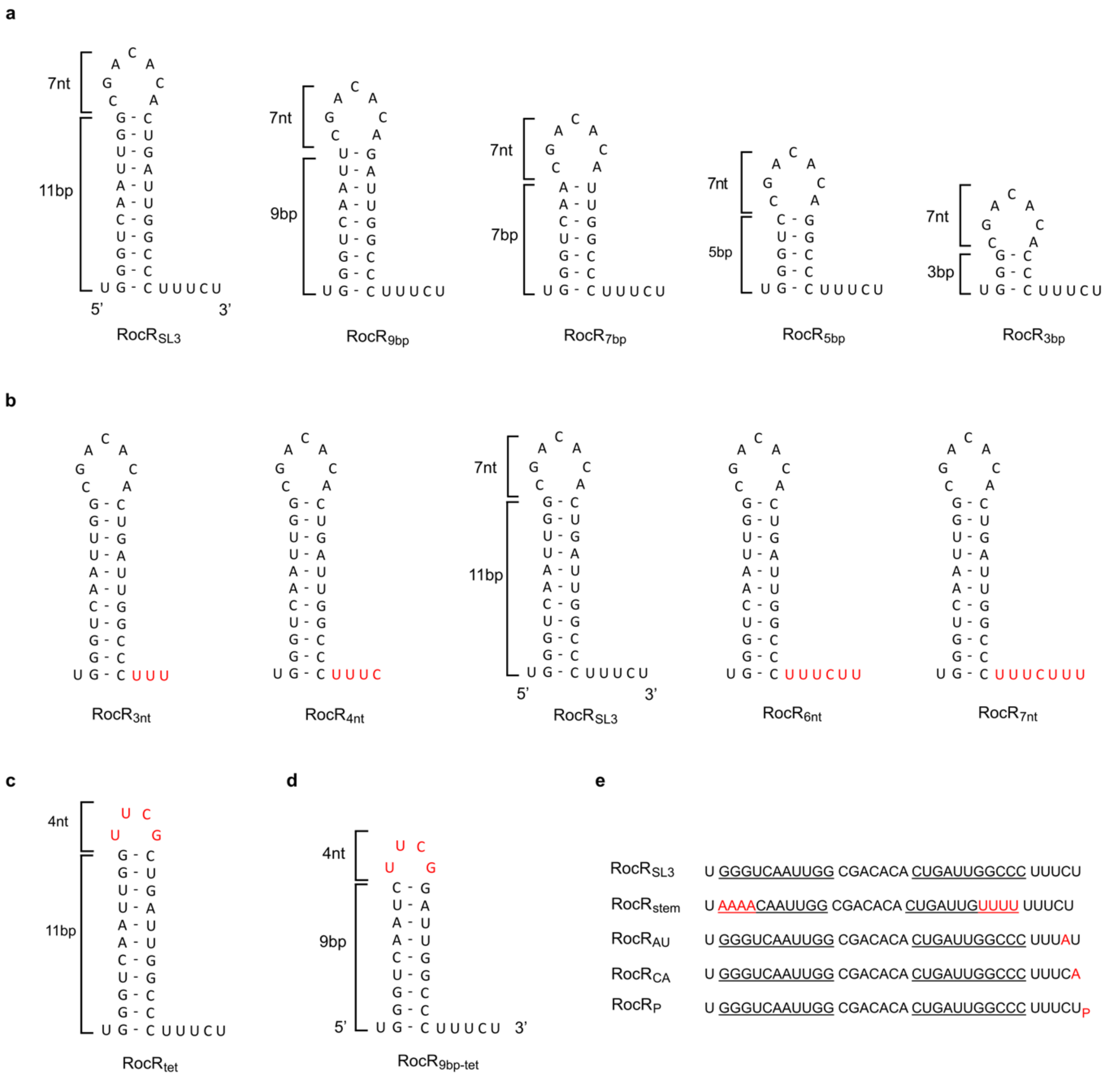

Supplementary Figure 1. Various RNA constructs used in RocC binding assays. (a) $RocR_{SL3}$ with different stem lengths. (b) $RocR_{SL3}$ with varying lengths of the tail. (c) $RocR_{SL3}$ with a tetraloop. (d) $RocR_{9bp\text{-}tet}$ crystallized with $RocC_{14\text{-}126}$. (e) Different substitution mutants of $RocR_{SL3}$ used for EMSA binding assays. Underlined regions show the predicted double-stranded stem. Red indicates substitutions from the original sequence.

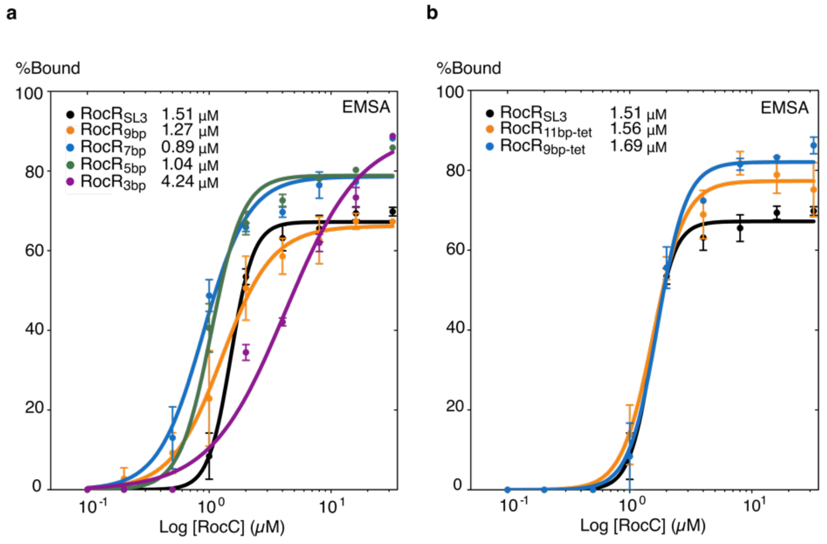

c

Construct	K_D (μM)
$RocR_{SL3}$	1.51 ± 0.07
$RocR_{3bp}$	4.24 ± 0.57
$RocR_{5bp}$	1.04 ± 0.05
$RocR_{7bp}$	0.89 ± 0.06
$RocR_{9bp}$	1.27 ± 0.15
$RocR_{3nt}$	ND
$RocR_{4nt}$	ND
$RocR_{6nt}$	ND
$RocR_{7nt}$	ND
$RocR_{stem}$	2.76 ± 0.37
$RocR_{AU}$	0.66 ± 0.05
$RocR_{CA}$	3.21 ± 0.18
$RocR_{11bp\text{-}tet}$	1.56 ± 0.10
$RocR_{9bp\text{-}tet}$	1.69 ± 0.08

$RocC_{14\text{-}126}$

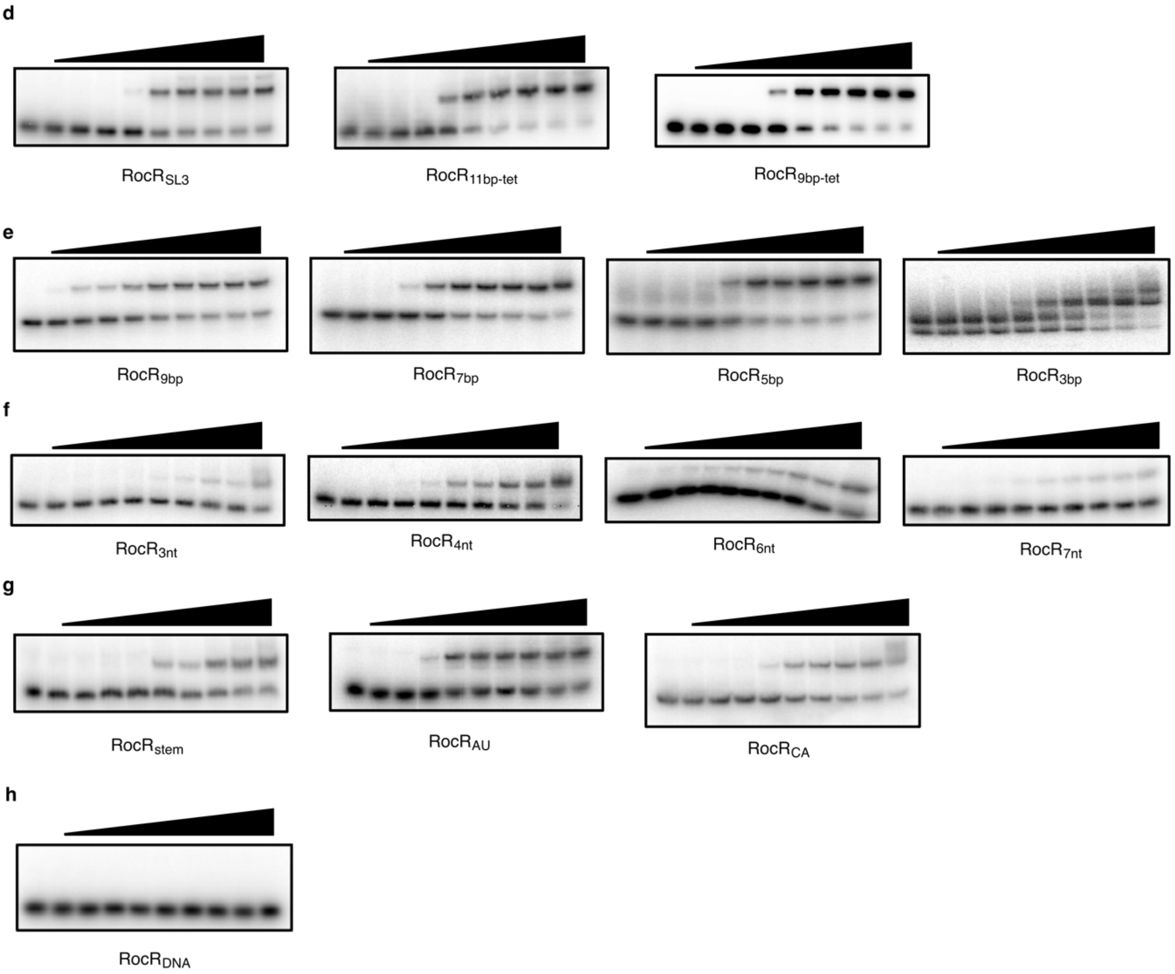

Supplementary Figure 2. Binding analyses of various $RocR_{SL3}$ mutants with $RocC_{14\text{-}126}$ using EMSA. (a) EMSA binding assay for $RocC_{14\text{-}126}$ with 5' radiolabeled $RocR_{SL3}$ with different stem lengths. The error bars are standard error of the mean (SEM) (n = 3). (b) EMSA binding assay for $RocC_{14\text{-}126}$ with 5' radiolabeled $RocR_{SL3}$ with different loop sizes. The error bars are the standard error of the mean (SEM) (n = 3). (c) Table of EMSA determined binding affinities between $RocC_{14\text{-}126}$ and the $RocR_{SL3}$ variants. (d-h) Representative EMSA gels with the indicated RocC and RocR constructs. Each experiment was repeated independently three times with similar results. The lanes represent 2-fold serial dilutions of RocC from 32 μM (10th lane) to 0.125 μM (2nd lane). (d) Comparison of the

effect of loop and stem composition on binding affinity. (e) Analysis of stem length on binding affinity. (f) Effect of 3’ ssRNA tail length on binding affinity. (g) Effect of substitution mutations on binding affinity. (h) A DNA version of RocR does not bind RocC. Original EMSA gel images are provided as a Source Data file.

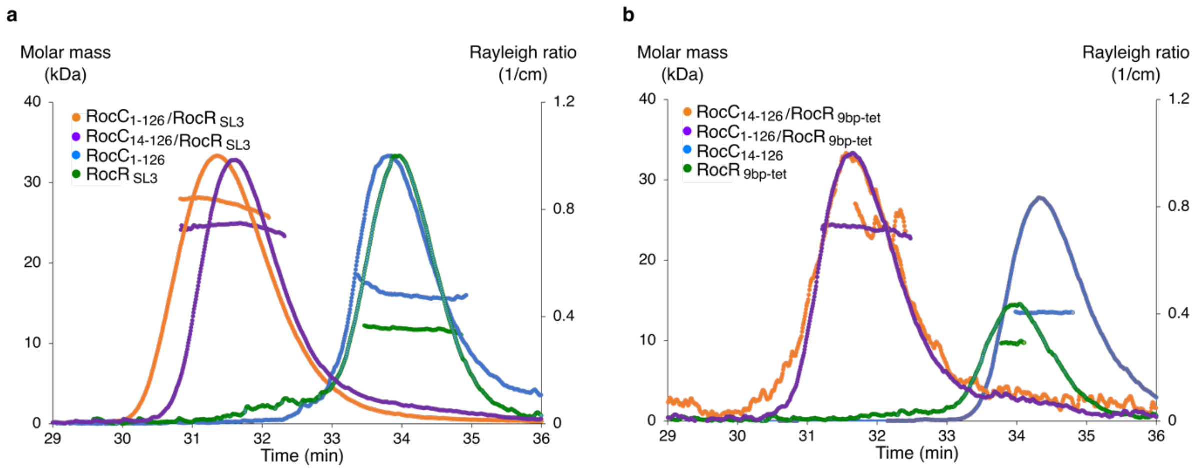

c

	Measured M.W. (kDa)	Theoretical M.W. (Da)	M_w/M_n	Oligomeric state
$RocC_{1\text{-}126}$	1.633×10^4 (± 0.036%)	15938.5	1.002 (± 0.023%)	Monomer
$RocR_{14\text{-}126}$	1.349×10^4 (±0.019%)	13252.32	1.000 (±0.028%)	Monomer
$RocR_{SL3}$	1.180×10^4 (± 0.013%)	11355.6	1.000 (± 0.019%)	Monomer
$RocR_{9bp\text{-}tet}$	9.661×10^3 (±7.121%)	9067.2	1.000 (±10.077%)	Monomer
$RocC_{1\text{-}126}/RocR_{SL3}$	2.743×10^4 (± 0.790%)	27294.1	1.001 (± 1.120%)	Heterodimer
$RocR_{1\text{-}126}/RocR_{9bp\text{-}tet}$	2.411×10^4 (±0.020%)	25005.72	1.000 (±0.028%)	Heterodimer
$RocR_{14\text{-}126}/RocR_{SL3}$	2.463×10^4 (±0.020%)	24607.92	1.000 (±0.028%)	Heterodimer
$RocR_{14\text{-}126}/RocR_{9bp\text{-}tet}$	2.422×10^4 (±18.863%)	22319.52	1.002 (±26.606%)	Heterodimer

Supplementary Figure 3. Purification and analysis of RocC/RocR oligomerization using SEC-MALS. (a) and (b) SEC-MALS data for the indicated RocC and RocR molecules and related complexes showing both the scattering traces and mass determinations across the peaks. (c) Table showing the theoretical and experimentally determined masses for the different complexes. Source data are provided as a Source Data file.

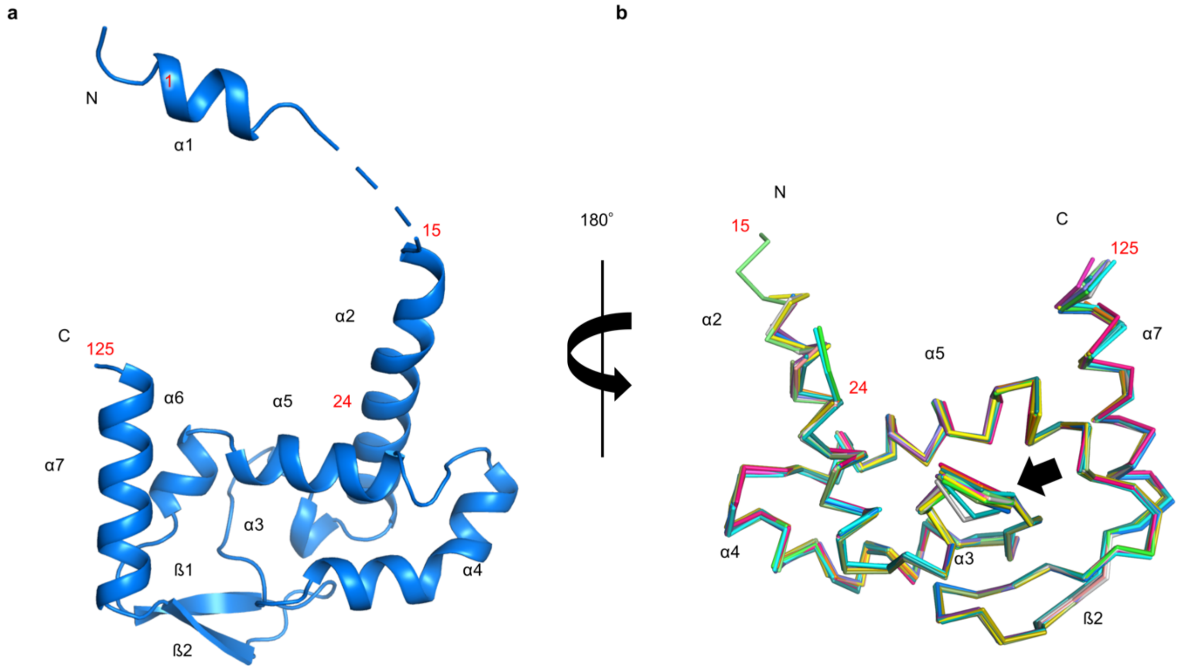

Supplementary Figure 4. Apo-RocC structure. Two apo-protein crystal structures, $RocC_{24\text{-}126}$ and $RocC_{1\text{-}126}$ were determined at 2.1 Å and 2.0 Å, respectively (see Methods, Supplementary Table 1). The $RocC_{1\text{-}126}$ crystal contains 9 protomers in the asymmetric unit, while the $RocC_{24\text{-}126}$ contains a single protomer in the asymmetric unit. $RocC_{24\text{-}126}$ constitutes a proteolytically stable core, and the crystal structure reveals a compact and well-folded structure. $RocC_{1\text{-}126}$ (a) has the same core structure, however the 1-23 segment adopts a flexible extended structure which is partially visualized in 2 of the 9 protomers in the asymmetric unit, suggesting that residues 1-7 have the potential to adopt a helical conformation. The core region $RocC_{24\text{-}126}$ is quite rigid, with an RMSD of 0.29 Å between $RocC_{1\text{-}126}$ and $RocC_{24\text{-}126}$ structures. The only region of significant flexibility is in a single loop linking α2 and α4. (a) Cartoon view of apo-$RocC_{1\text{-}126}$ with secondary structure elements and key residue positions labeled. (b) Cα traces of the aligned 9 $RocC_{1\text{-}126}$ protomers and single $RocC_{24\text{-}126}$ protomers, as well as the 10 copies of $RocC_{24\text{-}126}$ in the RocC/RocR crystallographic asymmetric unit. The view is rotated 180° relative to (a), and the black arrow indicates the conformationally flexible loop.

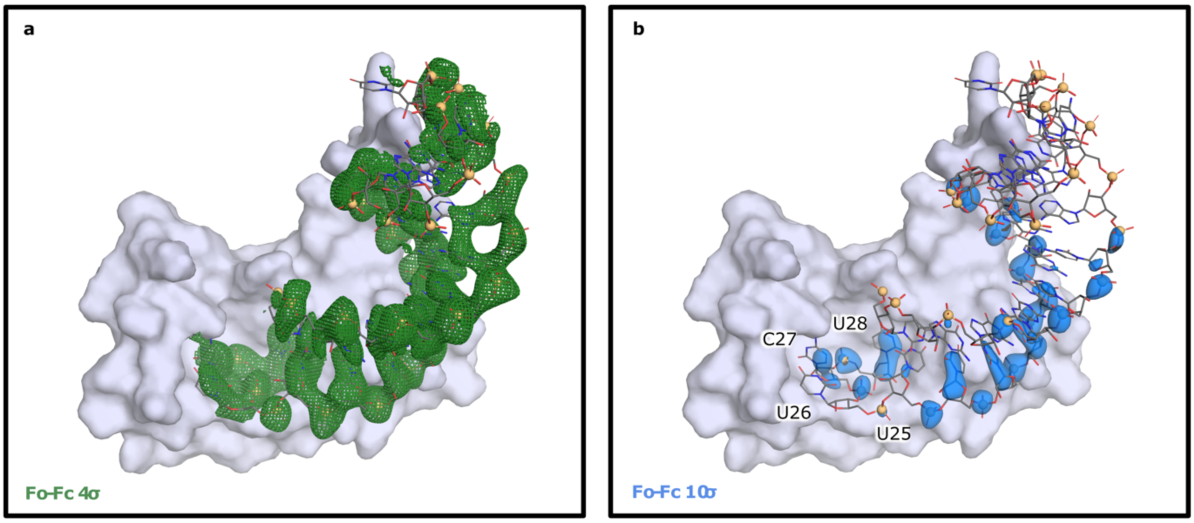

Supplementary Figure 5. Difference electron density used to build the $RocC_{14-126}$/$RocR_{9bp\text{-}tet}$ complex. The RocC/RocR crystal structure was initially solved by molecular replacement using the high resolution structure of $RocC_{14-126}$. Displayed is 4-fold averaged Fo-Fc density at 3.2 Å phased with the 10 RocC protomers in the asymmetric unit at 4 σ cutoff (green mesh, panel a) or 10 σ cutoff (blue surface, panel b) with the protein model displayed as a grey surface. The higher σ cutoff was used to help resolve the higher density phosphate groups from the bases and sugars in the RNA. The 4 nucleotides in the 3' tail are labeled in (b).

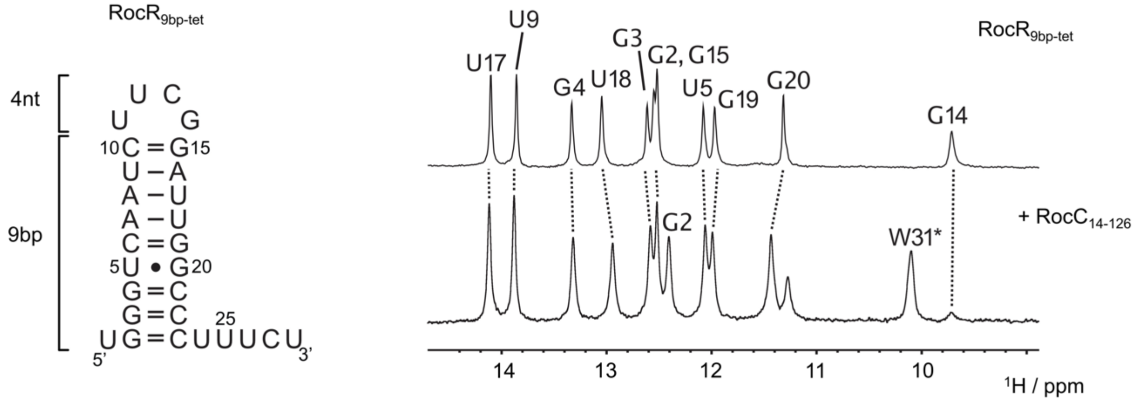

Supplementary Figure 6. ^{1}H NMR spectra of the imino region of RocR$_{SL3}$ 28nt RNA and the RocR$_{SL3}$/RocC$_{14\text{-}126}$ complex. The imino spectra indicate that the base-pairing pattern is the same for the free RNA and the complex. The unidentified signal at 11.3 ppm could not be connected to any imino residue but is believed to be a result of a specific non-Watson-Crick interaction either between protein and RNA or within the RNA, present only in the RNA-protein complex. W31* indicates the resonance for the side chain NH of Trp31.

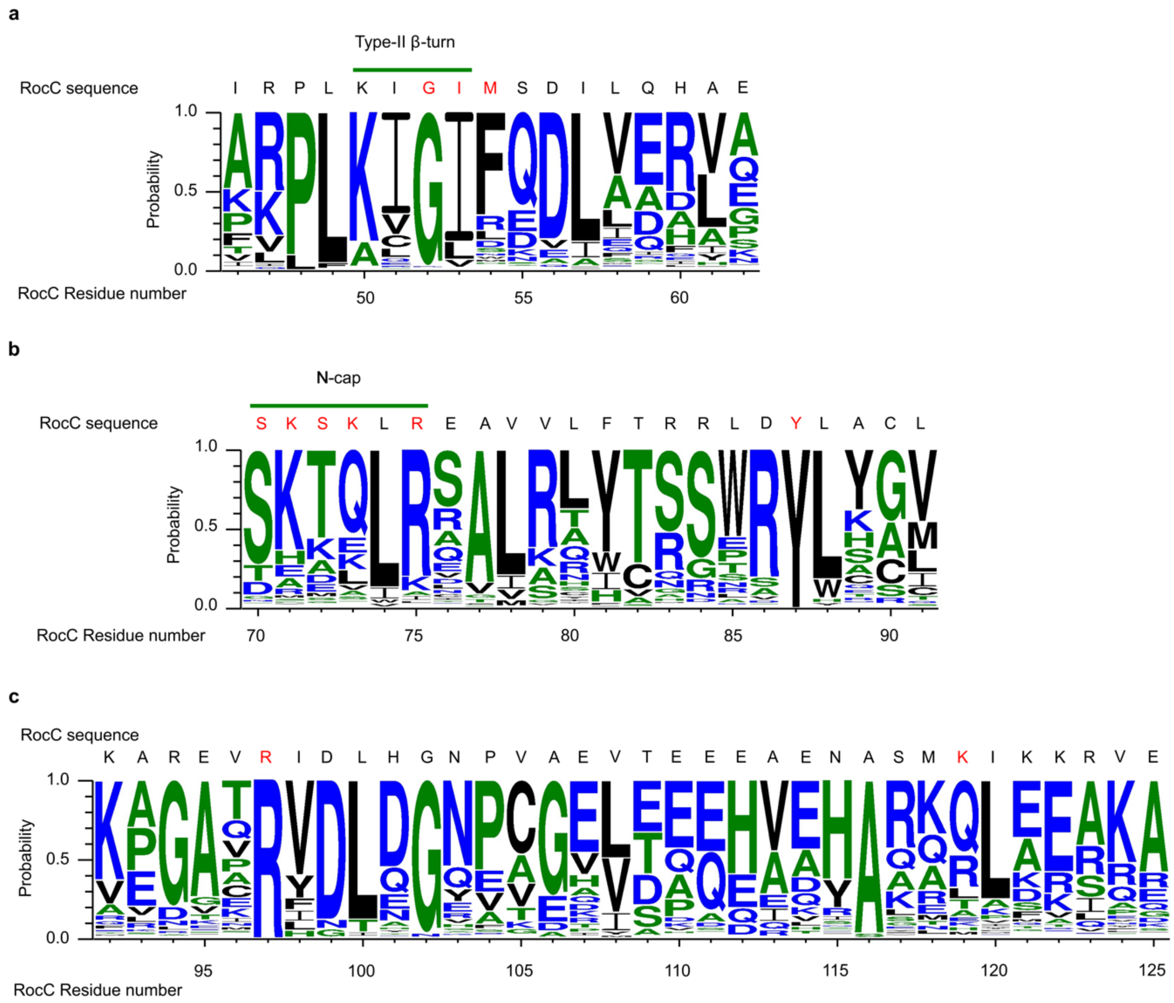

Supplementary Figure 7. Graphical representation of sequence conservation in 674 ProQ/FinO domain-containing proteins[1] using WebLogo3[2]. Red letters indicate the residues in contact with RocR$_{9bp\text{-}tet}$. (a) Sequence logo near the β-hairpin-α-helix motif. (b) Sequence logo near the N-cap motif. (c) Sequence logo for the C-terminal region of the RocC ProQ/FinO domain.

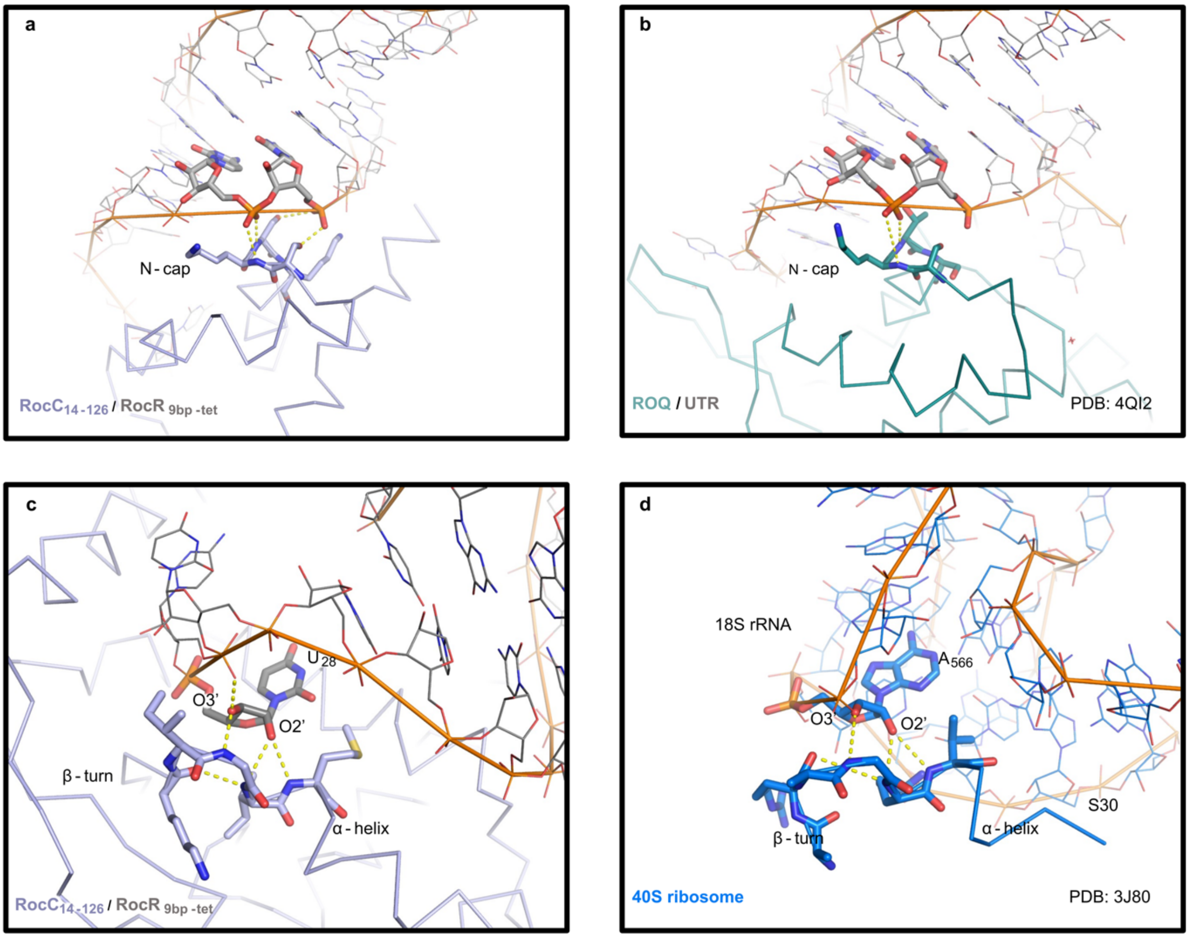

Supplementary Figure 8. RNA recognition motifs in RocC are found in other RNA binding proteins. (a) The N-cap motif of RocC recognizes phosphate groups along a single strand of the RocR hairpin stem. (b) A similar N-cap motif in the ROQ domain of the mammalian Roquin protein recognizes the RNA phosphate backbone in a similar manner. Hydrogen bonds between the N-cap and the RNA phosphates are indicated with dashed bonds. Key residues involved in the interactions are shown as sticks. (c) The β-turn-α-helix motif in RocC recognizes the 3' terminal nucleotide of RocR. (d) A similar β-turn-α-helix motif is found in the eukaryotic 40S ribosome between ribosomal protein eS30 and the 18S rRNA. In both cases, the O2' and O3' atoms of the C3'-endo ribose are hydrogen-bonded to the motif in similar ways.

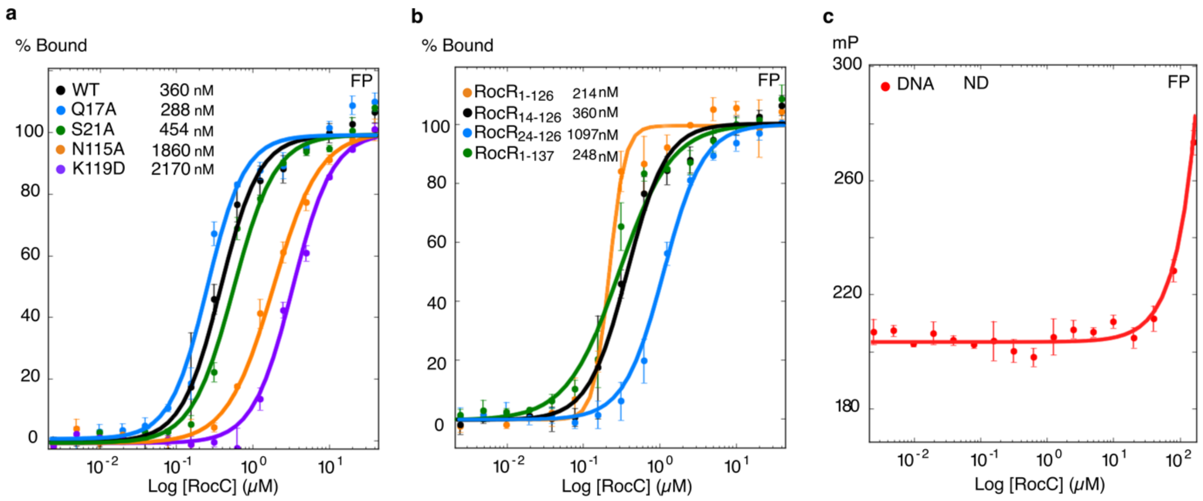

d

RocC	Mutant	K_D (μM)	Standard error (μM)	p-value
1-126	WT	0.214	0.007	0.1381
24-126	WT	1.097	0.019	<0.0001
1-137	WT	0.248	0.017	N/A
14-126	WT	0.36	0.03	0.0314
14-126	K15D	0.298	0.014	0.1344
14-126	Q17A	0.288	0.019	0.1125
14-126	K18D	0.213	0.009	0.0094
14-126	S21A	0.454	0.016	0.0506
14-126	I51A	0.3502	0.0032	0.7616
14-126	S70A	1.17	0.07	0.0004
14-126	K71D	4.2	0.3	0.0002
14-126	S72A	0.64	0.05	0.0086
14-126	K73D	4.4	0.1	<0.0001
14-126	R75D	ND	ND	ND
14-126	T82A	0.286	0.012	0.0838
14-126	R83D	1.06	0.05	0.0003
14-126	R97M	ND	ND	ND
14-126	N115A	1.86	0.07	<0.0001
14-126	K119D	2.17	0.05	<0.0001
14-126	S70/72A	ND	ND	ND

e

Protein	RNA Mutant	K_D (μM)	Standard error (μM)
$RocC_{14-126}$	DNA	ND	ND
$RocC_{14-126}$	$RocR_{3nt}$	ND	ND
$RocC_{14-126}$	$RocR_{5nt}$	0.374	0.029
$RocC_{14-126}$	$RocR_{8nt}$	ND	ND
$FinO_{45-186}$	$RocR_{3nt}$	2.02	0.11
$FinO_{45-186}$	$RocR_{5nt}$	0.903	0.078
$FinO_{45-186}$	$RocR_{8nt}$	1.03	0.82
$ProQ_{1-130}$	$RocR_{3nt}$	0.95	0.12
$ProQ_{1-130}$	$RocR_{5nt}$	0.289	0.018
$ProQ_{1-130}$	$RocR_{8nt}$	1.469	0.081

Supplementary Figure 9. Effects of RocC point mutations on RNA binding *in vitro* using FP. (a-b) FP binding assays for 5' FAM-labeled $RocR_{SL3}$ with different RocC* (n = 3). The $RocC_{14-126}$ point mutations are presented in (a) and the N-terminal truncations are presented in (b). The error bars are standard error of the mean (SEM). (c) FP binding assays for 5' FAM-labeled SL3 DNA with $RocC_{14-126}$ (n = 3). (d) and (e) Tables of K_D values derived from FP binding assays. ND indicates a K_D could not be determined because binding saturation was not achieved. p-values indicated here compare K_D of the $RocC_{14-126}$ point mutations to the WT. We considered a p-value < 0.05 to be statistically significant. The p-values come from using an equal variance (independent) one-sided t-test, which was used because data from each mutant is independent. No individual measurements were identified as outliers and therefore no individual measurements were discarded when calculating K_Ds. Source data are provided as a Source Data file.

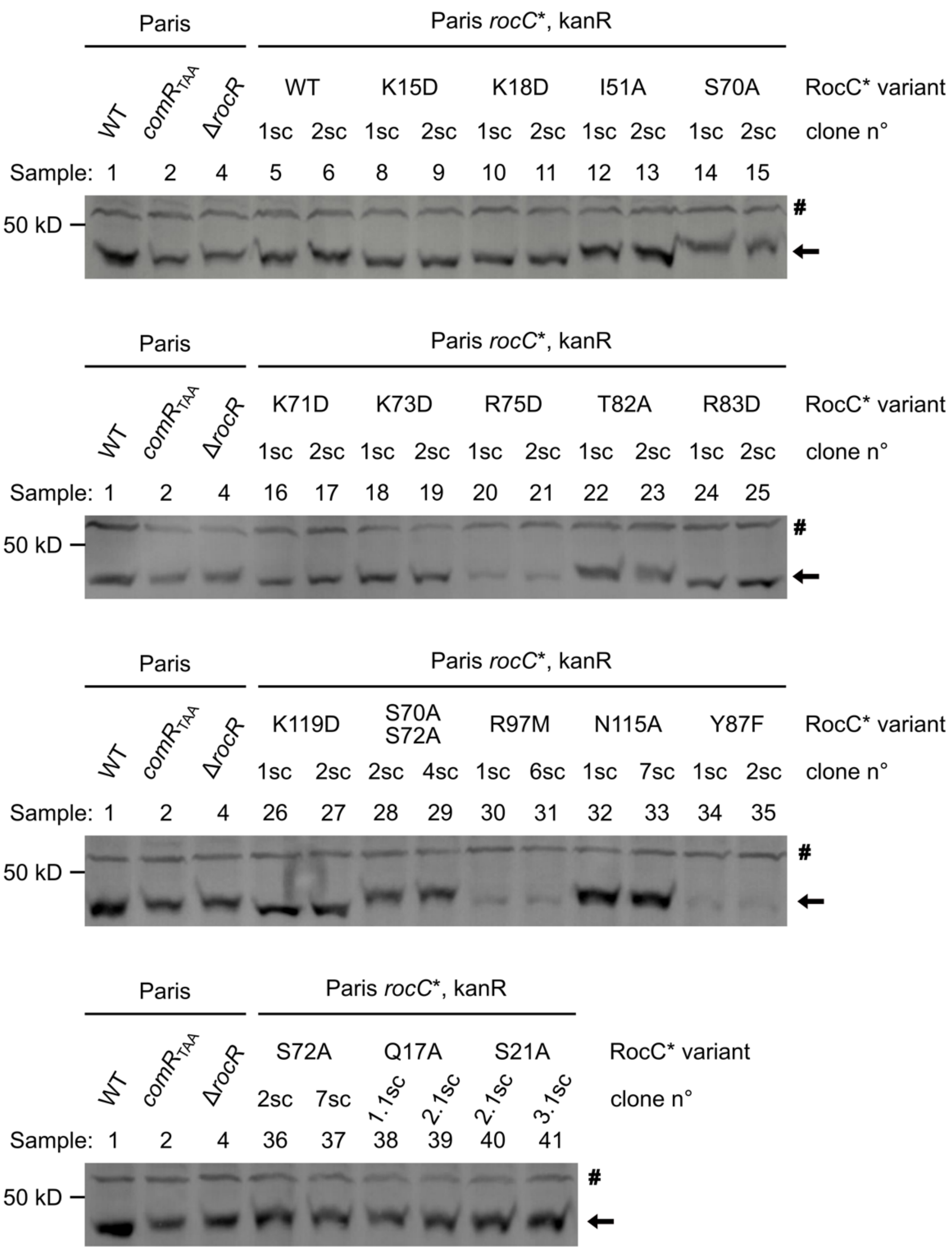

Supplementary Figure 10. Detection of RocC* variants *in vivo*. Western-blot analysis of RocC and RocC* variants in the Paris WT strain, the Δ*rocR* and $comR_{TAA}$ hypercompetent strains and the Paris *rocC*, kanR strain and its derivatives bearing a mutated allele of *rocC* instead of the WT allele. The strains were grown up to OD_{600}~1 in AYE medium @ 37°C. Samples of $1.5x10^8$ cells were analysed by SDS-PAGE and Western-blot using polyclonal anti-RocC antibodies. The symbol # indicates a cross-reactive band that can be used as a loading control, the arrow points to the bands corresponding to the RocC* proteins. This experiment was done at least twice on 2 independent clones of each mutant. Source data are provided as a Source Data file.

Supplementary Table 1. Data collection and statistics for RocC structure determination.

	$\mathbf{RocC_{24\text{-}126}}$ **(PDB ID: 7RGS)**	$\mathbf{RocC_{1\text{-}126}}$ **(PDB ID: 7RGT)**	$\mathbf{RocC_{14\text{-}126}/RocR_{9bp\text{-}tet}}$ **(PDB ID: 7RGU)**
**Data collection**			
Space group	$P2_12_12$	C2	$P2_12_12_1$
Cell dimensions			
a, b, c (Å)	55.56, 81.77, 49.42	139.01, 100.30, 100.72	87.81, 135.94, 156.76
α, β, γ (°)	90.00, 90.00, 90.00	90.00, 107.57, 90.00	90.00, 90.00, 90.00
Resolution (Å)	50.00-2.10 (2.14-2.10)	50.00-2.02 (2.05-2.02)	50.00-3.20 (3.31-3.20)
R_{meas}	0.134 (0.565)	0.123 (1.186)	0.334 (0.883)
I/σ (I)	35.70 (7.33)	17.43 (1.50)	4.98 (1.67)
Completeness (%)	97.9 (91.8)	99.7 (95.6)	91.4 (92.0)
Redundancy	36.5 (28.7)	6.2 (4.7)	4.4 (4.5)
**Refinement**			
Resolution range (Å)	49.42-2.10 (2.31-2.10)	45.30-2.02 (2.10-2.02)	48.77-3.20 (3.31-3.20)
No. reflections	13,164 (1,257)	94,408 (9,025)	28,975 (2,864)
R_{work}/R_{free}	0.161 (0.170)/0.238 (0.223)	0.193 (0.281)/0.213 (0.308)	0.219 (0.243)/0.271 (0.333)
No. atoms	2,087	8,197	20,397
Protein	1,724	7,669	17,113
RNA/ligand	-	45 (Ligand)	3,284 (RNA)
Water	363	483	-
B-factors			
Protein	25.1	67.7	44.8
RNA/ligand	-	65.2 (Ligand)	71.0 (RNA)
Water	33.7	52.2	-
r.m.s deviations			
Bond lengths (Å)	0.006	0.005	0.002
Bond angles (°)	0.74	0.71	0.59

Supplementary Table 2. Bacterial strains, plasmids and oligonucleotides used in this study

**Strains**	**Relevant genotype**	**Reference**
L. pneumophila		
**Paris WT**	Paris Outbreak isolate CIP107629	CNR Lyon
**Paris** ***rocC***$_{TAA}$	Paris; *rocC*$_{TAA}$ (previously noted *lpp0148*$_{TAA}$)	[3]
**Paris Δ*rocC::MK***	Paris; *rocC::(lacIq*, P_{tac}-*mazF*, *nptII*); KanR, $IPTG^S$	[3]
**Paris *ΔrocR***	Paris; *ΔrocR*	[1]
**Paris *rocC, kan***	Paris transformed by pLFP01; *rocC_lpp0149*::(*nptII*); KanR	This study
**Paris *rocC*$_{K15D}$*, kan***	Paris transformed by pLLA96; *rocC*$_{K15D}$*_lpp0149*::(*nptII*); KanR	This study
**Paris *rocC*$_{Q17A}$*, kan***	Paris transformed by pLLA112; *rocC*$_{Q17A}$*_lpp0149*::(*nptII*); KanR	This study
**Paris *rocC*$_{K18D}$*, kan***	Paris transformed by pLLA97; *rocC*$_{K18D}$*_lpp0149*::(*nptII*); KanR	This study
**Paris *rocC*$_{S21A}$*, kan***	Paris transformed by pLLA113; *rocC*$_{S21A}$*_lpp0149*::(*nptII*); KanR	This study
**Paris *rocC*$_{I51A}$*, kan***	Paris transformed by pLLA98; *rocC*$_{I51A}$*_lpp0149*::(*nptII*); KanR	This study
**Paris *rocC*$_{S70A}$*, kan***	Paris transformed by pLLA99; *rocC*$_{S70A}$*_lpp0149*::(*nptII*); KanR	This study
**Paris *rocC*$_{S72A}$*, kan***	Paris transformed by pLLA111; *rocC*$_{S72A}$*_lpp0149*::(*nptII*); KanR	This study
**Paris *rocC*$_{S70A-S72A}$*, kan***	Paris transformed by pLLA106; *rocC*$_{S70A-S72A}$*_lpp0149*::(*nptII*); KanR	This study
**Paris *rocC*$_{K71D}$*, kan***	Paris transformed by pLLA100; *rocC*$_{K71D}$*_lpp0149*::(*nptII*); KanR	This study
**Paris *rocC*$_{K73D}$*, kan***	Paris transformed by pLLA101; *rocC*$_{K73D}$*_lpp0149*::(*nptII*); KanR	This study
**Paris *rocC*$_{R75D}$*, kan***	Paris transformed by pLLA102; *rocC*$_{R75D}$*_lpp0149*::(*nptII*); KanR	This study
**Paris *rocC*$_{T82A}$*, kan***	Paris transformed by pLLA103; *rocC*$_{T82A}$*_lpp0149*::(*nptII*); KanR	This study
**Paris *rocC*$_{R83D}$*, kan***	Paris transformed by pLLA104; *rocC*$_{R83D}$*_lpp0149*::(*nptII*); KanR	This study
**Paris *rocC*$_{Y87F}$*, kan***	Paris transformed by pLFP08; *rocC*$_{Y87F}$*_lpp0149*::(*nptII*); KanR	This study
**Paris *rocC*$_{R97M}$*, kan***	Paris transformed by pLLA109; *rocC*$_{R97M}$*_lpp0149*::(*nptII*); KanR	This study
**Paris *rocC*$_{N115A}$*, kan***	Paris transformed by pLLA110; *rocC*$_{N115A}$*_lpp0149*::(*nptII*); KanR	This study
**Paris *rocC*$_{K119D}$*, kan***	Paris transformed by pLLA105; *rocC*$_{K119D}$*_lpp0149*::(*nptII*); KanR	This study
**Paris *rocCΔN14***	Paris Δ*rocC::MK* transformed by PCR DE_1; *rocCΔN14*	This study
**Paris *rocCΔN19***	Paris Δ*rocC::MK* transformed by PCR DE_3; *rocCΔN19*	This study
**Paris *rocCΔN24***	Paris Δ*rocC::MK* transformed by PCR DE_5; *rocCΔN24*	This study
E. coli		
**DH5α, λpir**	F^-, *sup*E44, Δ*lac*U169 (Φ*lacZ*ΔM15), *recA*1, *endA*1, *hsdR*17, *thi-1*, *gyrA*96, *relA*1, λ*pir* lysogen	Laboratory strain collection
BL21-Gold (DE3)	*F^- ompT hsdS(r_B^- m_B^-) dcm^+ Tet^r gal λ(DE3) endA Hte*	Laboratory strain collection
**Plasmids**	**Relevant genotype**	**Reference**
**pET-47b(+) RocC**$_{1-230}$	pET-47b(+) derivative; IPTG-inducible production of His-(HRV-3C)-Lpp0148$_{1-230}$ (Paris); KanR	[1]
**pGEM-ihfB::Kan**	pGEM-T Easy; *ihfB*::*nptII*; AmpR, KanR	[3]
**pGEM-MK**	pGEM-T Easy; FRT-(*lacIq*, P_{tac}-*mazF*, *nptII*)-FRT; AmpR, KanR	[4]
**pGEMPKD4**	pGEM-T Easy; FRT- *nptII* -FRT; AmpR, KanR	[1]
**pGEX-6P-1**	pBR322 derivative; IPTG-inducible production of GST-(HRV-3C)-protein; used for in vitro production of RocC mutants; AmpR	Cytiva 28-9546-48
**pLFP01**	pGEM-T Easy; *rocC_lpp0149*::(*nptII*); AmpR, KanR	This study
**pLFP08**	pLFP01 derivative; *rocC*$_{Y87F}$*_lpp0149*::(*nptII*); AmpR, KanR	This study
**pLLA96**	pLFP01 derivative; *rocC*$_{K15D}$*_lpp0149*::(*nptII*); AmpR, KanR	This study
**pLLA97**	pLFP01 derivative; *rocC*$_{K18D}$*_lpp0149*::(*nptII*); AmpR, KanR	This study
**pLLA98**	pLFP01 derivative; *rocC*$_{I51A}$*_lpp0149*::(*nptII*); AmpR, KanR	This study
**pLLA99**	pLFP01 derivative; *rocC*$_{S70A}$*_lpp0149*::(*nptII*); AmpR, KanR	This study
**pLLA100**	pLFP01 derivative; *rocC*$_{K71D}$*_lpp0149*::(*nptII*); AmpR, KanR	This study
**pLLA101**	pLFP01 derivative; *rocC*$_{K73D}$*_lpp0149*::(*nptII*); AmpR, KanR	This study
**pLLA102**	pLFP01 derivative; *rocC*$_{K75D}$*_lpp0149*::(*nptII*); AmpR, KanR	This study
**pLLA103**	pLFP01 derivative; *rocC*$_{T82A}$*_lpp0149*::(*nptII*); AmpR, KanR	This study
**pLLA104**	pLFP01 derivative; *rocC*$_{R83D}$*_lpp0149*::(*nptII*); AmpR, KanR	This study

**pLLA105**	pLFP01 derivative; *rocC*$_{K119D}$_*lpp0149*::(*nptII*); AmpR, KanR	This study
**pLLA106**	pLFP01 derivative; *rocC*$_{S70A-S72A}$_*lpp0149*::(*nptII*); AmpR, KanR	This study
**pLLA109**	pLFP01 derivative; *rocC*$_{R97M}$_*lpp0149*::(*nptII*); AmpR, KanR	This study
**pLLA110**	pLFP01 derivative; *rocC*$_{N115A}$_*lpp0149*::(*nptII*); AmpR, KanR	This study
**pLLA111**	pLFP01 derivative; *rocC*$_{S72A}$_*lpp0149*::(*nptII*); AmpR, KanR	This study
**pLLA112**	pLFP01 derivative; *rocC*$_{Q17A}$_*lpp0149*::(*nptII*); AmpR, KanR	This study
**pLLA113**	pLFP01 derivative; *rocC*$_{S21A}$_*lpp0149*::(*nptII*); AmpR, KanR	This study

**Cloning primers**	**Sequence (5' to 3')**
**lpp0148_P1**	GCCGTTTTAAATCGGCCAGAAAG
**lpp0148_P2**	ggcccaattcgccctatagtgagtcgCAGGCAAGCCAGATAATCAAGAC
**lpp0148_P3**	gggtttgctcgggtcggtggcataTGAAAGCTCGTGAAGTCCGTATCG
**lpp0148_P4**	CTTCCCCCTAAAAATCAGGATGTC
**lpp0148RM_P3**	GAACTAAGGAGGATATTCATATGGACCATGGCTGTTTATTTCTCCATTATGGGCTG
**lpp0148RM_R**	GGAACTTCGAAGCAGCTCCAGCCTACACAATCTGCTGAAAATAATGTCGCTATTCGC
**lpp0149pX3_R**	ATCACCCGGGCTAAGGTTGTTTTAAAATCATAAAGTAAGC
**MazFk7-F**	CGACTCACTATAGGGCGAATTGGGCCGCTTTCCAGTCGGGAAACCTG
**MazF-R**	CATATGCCACCGACCCGAGCAAACCCGAAGAAGTTGTCCATATTGGCCAC
**pKD4_P1**	GATTGTGTAGGCTGGAGCTGCTTCG
**pKD4_P2**	GCCATGGTCCATATGAATATCCTCC

Supplementary Table 3. Oligonucleotides used to create pLFP01 derivatives with punctual *rocC* mutants

Mutation	Forward primer		Obtained plasmid
	name	sequence (5'-3') (mutations are in lowercase)	
**K15D**	LA161_rocCk15d_F	GAACCGCTGTCATCAATgAcGCACAAAAAAATCAATCC	pLLA96
**Q17A**	LA189_rocCq17a_F	CCGCTGTCATCAATAAAGCAgcAAAAAATCAATCCAAGCGCG	pLLA112
**K18D**	LA163_rocCk18d_F	CATCAATAAAGCACAAgAcAATCAATCCAAGCGCG	pLLA97
**S21A**	LA191_rocCs21a_F	CAATAAAGCACAAAAAAATCAAgCCAAGCGCGCGCGATCTG	pLLA113
**I51A**	LA165_rocCi51a_F	GTATTCGGCCATTAAAGgcTGGTATTATGTCTGATATATTG	pLLA98
**S70A**	LA167_rocCs70a_F	GCAGAGCAAGTTGGAGTTgCTAAAAGCAAATTAAGGGAAGC	pLLA99
**S72A**	LA187_rocCs72a_F	GCAAGTTGGAGTTTCTAAAgcCAAATTAAGGGAAGCTGTTG	pLLA111
**S70A, S72A**	LA181_rocCs70.72a_F	GCAGAGCAAGTTGGAGTTgCTAAAgcCAAATTAAGGGAAGCTGTTG	pLLA106
**K71D**	LA169_rocCk71d_F	GAGCAAGTTGGAGTTTCTgAcAGCAAATTAAGGGAAGCTG	pLLA100
**K73D**	LA171_rocCk73d_F	GTTGGAGTTTCTAAAAGCgAcTTAAGGGAAGCTGTTGTTC	pLLA101
**R75D**	LA173_rocCr75d_F	GAGTTTCTAAAAGCAAATTAgacGAAGCTGTTGTTCTTTTTAC	pLLA102
**T82A**	LA175_rocCt82a_F	GGAAGCTGTTGTTCTTTTTgCCCGTCGTCTTGATTATCTG	pLLA103
**R83D**	LA177_rocCr83d_F	GCTGTTGTTCTTTTTACCgaTCGTCTTGATTATCTGGCTTG	pLLA104
**Y87F**	FP7_paris-rocC35_F	TTTTACCCGTCGTCTTGATTTTCTGGCTTGCCTGAAAGCTC	pLFP08
**R97M**	LA183_rocCr97m_F	TGAAAGCTCGTGAAGTCatgATCGATTTGCATGGAAATCC	pLLA109
**N115A**	LA185_rocCn115a_F	CTGAGGAAGAAGCGGAGgcTGCTTCCATGAAAATTAAAAAACGCG	pLLA110
**K119D**	LA179_rocCk119d_F	GAAGCGGAGAATGCTTCCATGgAcATTAAAAAACGCGTGGAAAAG	pLLA105
**Mutation**	**Reverse primer**		**Obtained plasmid**
	**name**	**sequence (5'-3') (mutations are in lowercase)**	
**K15D**	LA162_rocCk15d_R	GATTTTTTTGTGCgTcATTGATGACAGCGGTTC	pLLA96
**Q17A**	LA190_rocCq17a_R	CGCGCTTGGATTGATTTTTTgcTGCTTTATTGATGACAGCGG	pLLA112
**K18D**	LA164_rocCk18d_R	CGCGCTTGGATTGATTgTcTTGTGCTTTATTGATG	pLLA97
**S21A**	LA192_rocCs21a_R	CAGATCGCGCGCGCTTGGcTTGATTTTTTTGTGCTTTATTG	pLLA113
**I51A**	LA166_rocCi51a_R	CAATATATCAGACATAATACCAgcCTTTAATGGCCGAATAC	pLLA98
**S70A**	LA168_rocCs70a_R	GCTTCCCTTAATTTGCTTTTAGcAACTCCAACTTGCTCTGC	pLLA99
**S72A**	LA188_rocCs72a_R	CAACAGCTTCCCTTAATTTGgcTTTAGAAACTCCAACTTGC	pLLA111
**S70A, S72A**	LA182_rocCs70.72a_R	CAACAGCTTCCCTTAATTTGgcTTTAGcAACTCCAACTTGCTCTGC	pLLA106
**K71D**	LA170_rocCk71d_R	CAGCTTCCCTTAATTTGCTgTcAGAAACTCCAACTTGCTCTG	pLLA100
**K73D**	LA172_rocCk73d_R	GAACAACAGCTTCCCTTAAgTcGCTTTTAGAAACTCCAACTTG	pLLA101
**R75D**	LA174_rocCr75d_R	GTAAAAAGAACAACAGCTTCgtcTAATTTGCTTTTAGAAACTC	pLLA102
**T82A**	LA176_rocCt82a_R	CAGATAATCAAGACGACGGGcAAAAAGAACAACAGCTTCC	pLLA103
**R83D**	LA178_rocCr83d_R	CAAGCCAGATAATCAAGACGAtcGGTAAAAAGAACAACAGC	pLLA104
**Y87F**	FP7_paris-rocC35_R	GAGCTTTCAGGCAAGCCAGAAAATCAAGACGACGGGTAAAA	pLFP08
**R97M**	LA184_rocCr97m_R	GATTTCCATGCAAATCGATcatGACTTCACGAGCTTTC	pLLA109
**N115A**	LA186_rocCn115a_R	CGTTTTTTAATTTTCATGGAAGCAgcCTCCGCTTCTTCCTCAG	pLLA110
**K119D**	LA180_rocCk119d_R	CTTTTCCACGCGTTTTTTAATgTcCATGGAAGCATTCTCCGCTTC	pLLA105

Supplementary Table 4. DNA templates used for *in vitro* transcription

DNA template	Sequence (5’-3’)
**T7 RNAP promoter Rev**	TAATACGACTCACTATAGG
**RocR$_{SL3}$ Fwd**	AGAAAGGGCCAATCAGTGTGTCGCCAATTGACCCACCCTATAGTGAGTCGTATTA
**RocR$_{SL3}$ Rev**	TAATACGACTCACTATAGGGTGGGTCAATTGGCGACACACTGATTGGCCCTTTCT
**RocR$_{3nt}$ Fwd**	AAAGGGCCAATCAGTGTGTCGCCAATTGACCCACCCTATAGTGAGTCGTATTA
**RocR$_{3nt}$ Rev**	TAATACGACTCACTATAGGGTGGGTCAATTGGCGACACACTGATTGGCCCTTT
**RocR$_{4nt}$ Fwd**	GAAAGGGCCAATCAGTGTGTCGCCAATTGACCCACCTATAGTGAGTCGTATTA
**RocR$_{6nt}$ Fwd**	AAGAAAGGGCCAATCAGTGTGTCGCCAATTGACCCACCTATAGTGAGTCGTATTA
**RocR$_{7nt}$ Fwd**	AAAGAAAGGGCCAATCAGTGTGTCGCCAATTGACCCACCTATAGTGAGTCGTATTA
**RocR$_{stem}$ Fwd**	AGAAAAAAACAATCAGTGTGTCGCCAATTGTTTTACCTATAGTGAGTCGTATTA
**RocR$_{AU}$ Fwd**	ATAAAGGGCCAATCAGTGTGTCGCCAATTGACCCACCCTATAGTGAGTCGTATTA
**RocR$_{AU}$ Rev**	TAATACGACTCACTATAGGGTGGGTCAATTGGCGACACACTGATTGGCCCTTTAT
**RocR$_{CA}$ Fwd**	TGAAAGGGCCAATCAGTGTGTCGCCAATTGACCCACCTATAGTGAGTCGTATTA
**RocR$_{9bp}$ Fwd**	AGAAAGGGCCAATCTGTGTCGAATTGACCCACCTATAGTGAGTCGTATTA
**RocR$_{7bp}$ Fwd**	AGAAAGGGCCAATGTGTCGTTGACCCACCTATAGTGAGTCGTATTA
**RocR$_{7bp}$ Rev**	TAATACGACTCACTATAGGTGGGTCAACGACACATTGGCCCTTTCT
**RocR$_{5bp}$ Fwd**	AGAAAGGGCCTGTGTCGGACCCACCTATAGTGAGTCGTATTA
**RocR$_{5bp}$ Rev**	TAATACGACTCACTATAGGTGGGTCCGACACAGGCCCTTTCT
**RocR$_{3bp}$ Fwd**	AGAAAGGGTGTGTCGCCCACCTATAGTGAGTCGTATTA
**RocR$_{3bp}$ Rev**	TAATACGACTCACTATAGGTGGGCGACACACCCTTTCT
**RocR$_{11bp\text{-}tet}$ Fwd**	AGAAAGGGCCAATCACCGAAGCAATTGACCCACCCTATAGTGAGTCGTATTA
**RocR$_{11bp\text{-}tet}$ Rev**	TAATACGACTCACTATAGGGTGGGTCAATTGCTTCGGTGATTGGCCCTTTCT
**RocR$_{9bp\text{-}tet}$ Fwd**	AGAAAGGGCCAATCCGAAGATTGACCCACCCTATAGTGAGTCGTATTA
**RocR$_{9bp\text{-}tet}$ Rev**	TAATACGACTCACTATAGGGTGGGTCAATCTTCGGATTGGCCCTTTCT

Fwd refers to forward strand, Rev refers to the complementary reverse strand.

Supplementary Table 5. Oligonucleotides used to create pGEX-6P-1 derivatives with truncation *rocC* mutants

Primer	Sequence (5’-3’)
**RocCdel1_Fwd_BamH1**	CTAGTCGGATCCATGAGAAAGCAGGCGCT
**RocCdel14_Fwd_BamH1**	TCAGGATCCAATAAAGCACAAAAAAATCAATCCAAGC
**RocCdel24_Fwd_BamH1**	CTGAATGGATCCGCGCGATCTGAC
**RocC126_Rev_Not1**	GACTAGGCGGCCGCTTACTTTTCCACGCGTTTTTTAATTTTC
**RocC137_Rev_Not1**	GACTAGGCGGCCGCTTATGCATTCACTTGTTTGCGAG

Supplementary Table 6. Oligonucleotides used to create pGEX-6P-1 derivatives with punctual *rocC* mutants

Mutation	Forward primer	Reverse primer
	**sequence (5'-3')**	**sequence (5'-3')**
**K15D**	TCAGGATCCAATGATGCACAAAAAAATCAATCCAAGC	GACTAGGCGGCCGCTTACTTTTCCACGCGTTTTTTAATTTTC
**Q17A**	TAAAGCAGCAAAAAATCAATCCAAGCGCG	TTGGATCCCAGGGGCCCC
**K18D**	TCAGGATCCAATAAAGCACAAGATAATCAATCCAAGC	GACTAGGCGGCCGCTTACTTTTCCACGCGTTTTTTAATTTTC
**S21A**	TCAGGATCCAATAAAGCACAAAAAAATCAAGCCAAGC	GACTAGGCGGCCGCTTACTTTTCCACGCGTTTTTTAATTTTC
**I51A**	GCCATTAAAGGCTGGTATTATGTCTGATATATTG	CGAATACGCAAAGAATTATC
**S70A**	AGTTGGAGTTGCTAAAAGCAAATTAAG	TGCTCTGCTTTTTCTGCATG
**S72A**	AGTTTCTAAAGCCAAATTAAGGGAAGCTGTTG	CCAACTTGCTCTGCTTTTTC
**S70A, S72A**	AAGCCAAATTAAGGGAAGCTGTTG	TAGCAACTCCAACTTGCTCTG
**K71D**	TGGAGTTTCTGATAGCAAATTAAGGG	ACTTGCTCTGCTTTTTCTG
**K73D**	TTCTAAAAGCGATTTAAGGGAAGCTGTTG	ACTCCAACTTGCTCTGCT
**R75D**	AAGCAAATTAGATGAAGCTGTTGTTCTTTTTAC	TTAGAAACTCCAACTTGC
**T82A**	TGTTCTTTTTGCCCGTCGTCTTG	ACAGCTTCCCTTAATTTGC
**R83D**	TCTTTTTACCGATCGTCTTGATTATCTGGC	ACAACAGCTTCCCTTAATTTG
**Y87F**	CGTCTTGATTTTCTGGCTTGC	ACGGGTAAAAAGAACAAC
**R97M**	TCGTGAAGTCATGATCGATTTGCATGGAAATCCAGTAGC	GCTTTCAGGCAAGCCAGA
**N115A**	AGAAGCGGAGGCTGCTTCCATGAAAATTAAAAAAC	TCCTCAGTAACCTCCGCT
**K119D**	TCAGGATCCAATAAAGCACAAAAAAATCAATCCAAGC	GACGCGGCCGCTTACTTTTCCACGCGTTTTTTAATATCCAT

SUPPLEMENTARY METHODS

Construction of plasmids and strains

Construction of L. pneumophila strains with mutated RocC, punctual mutants

We first created the pLFP01 plasmid. Using PCR (PrimeStarMax, Takara), we amplified *rocC* from the Paris WT strain using primer pair lpp0148_P1/lpp0148RM_R, a kanamycin resistance cassette (*nptII*) from the pGEMPKD4 using primer pair pKD4_P1/pKD4_P2 and lpp0149 (which follows *rocC* on the genome) from the Paris WT strain using primer pair lpp0148RM_P3/lpp0149pX3_R. The 3 PCR fragments were assembled by PCR overlap extension using primer pair lpp0148_P1/lpp0149pX3_R and cloned into the pGEM-T Easy vector (Promega). After transformation in *E. coli* DH5α, λpir, transformants were selected on LB plates containing kanamycin. The plasmid was verified by PCR and sequencing.

The plasmid pLFP01 [*rocC-lpp0149*::(*nptII*)] was submitted to site-directed mutagenesis to create the different punctual mutants of RocC. PCRs were done using primer pairs designed to change the desired amino acid (see Supplementary Table 3) on pLFP01 as template. PCR products were then digested by DpnI to remove the parental pLFP01 and transformed in *E. coli* DH5α λpir. Transformants were selected on LB plates with kanamycin. Plasmids were verified by PCR and sequencing.

Each plasmid was then used to transform *L. pneumophila* Paris $rocC_{TAA}$ by natural transformation. As these plasmids are non-replicative in *L. pneumophila*, the internalized molecules recombine with the chromosome via a double cross-over allowing the replacement of the $rocC_{TAA}$ allele with the mutated *rocC* allele. Transformants were selected on CYE plates with kanamycin. The *rocC* locus was verified by PCR and sequencing, and the presence of the protein was verified by Western-Blot (Supplementary Figure 10). For each mutant, 2 independent clones were kept and tested.

Construction of L. pneumophila strains with mutated RocC, Nterminal deletions

Markerless N-terminal deletions of *rocC* were constructed in two steps, taking advantage of the counterselectable MK cassette. This MK cassette bears a kanamycin resistance gene and the toxin-encoding *mazF* gene under the control of an IPTG-inducible promoter (*lacIq*, P_{tac}-*mazF*, *nptII*) and was cloned into the pGEM-T Easy vector (Promega) to create plasmid pGEM-MK [4]. Insertion of

MK in *rocC* can be selected on CYE plates with kanamycin after transformation of a composite PCR "upstream *rocC* - MK - downstream *rocC"*. Replacement of the *rocC*::MK allele by the *rocC*ΔNter alleles can be selected on CYE+IPTG after transformation of a composite PCR (upstream - *rocC*ΔNter – downstream). To make the Paris *rocC*::MK strain, the upstream (PCR A: lpp0148_P1 / lpp0148_P2, 2050 bp) and downstream (PCR C: lpp0148_P3 / lpp0148_P4, 2015 bp) regions of *rocC* were amplified from Paris WT chromosome with primers carrying 30-nt sequences complementary to the ends of the MK cassette (PCR B, 3177 bp amplified from plasmid pGEM-MK with primer pair MazFk7-F/MazF-R). The 3 fragments were joined by PCR overlap extension (PCR lpp0148_P1 / lpp0148_P4, 7190 bp) and used to transform *L. pneumophila* WT by natural transformation. Transformants were selected on CYE plates with kanamycin and tested for sensitivity to IPTG. Integration of the MK cassette at the correct locus was verified by PCR.

To obtain the markerless *rocC*ΔNter mutants, a second step was performed as follows. The upstream and *rocC* regions were amplified with primers carrying a 30-nt tail sequence designed to create different *rocC*ΔNter alleles (PCR D and PCR E, see Supplementary Table 7 for details). These PCRs were assembled by PCR overlap extension using primer pair lpp0148_P1 / lpp0148_P4 (4 kb) and used to transform the Δ*rocC*::MK strain. Transformants were selected on CYE plates with IPTG and tested for sensitivity to kanamycin. Proper replacement of the *rocC* allele was verified by PCR and sequencing, and the presence of the protein was verified by Western-Blot. For each mutant, 2 independent clones were kept and tested.

E. coli strains with plasmid allowing the production of RocC (truncated or mutated)*

RocC mutants used in this study were amplified from pET-47b(+) $RocC_{1\text{-}230}$ [1] using appropriate primers purchased from IDT (see Supplementary Table 5 and 6) and the Phusion high-fidelity DNA polymerase (NEB). The resulting *rocC** gene fragments were digested and ligated between the BamH1 and Not1 sites in pGEX-6P-1 such as to produce a GST-(HRV-3C)-RocC* protein. Ligated DNAs were transformed into E. coli DH5α (Invitrogen). Transformants were selected on LB plates with ampicillin. Obtained plasmids were verified using Sanger sequencing.

SEC-MALS

All samples were prepared in SEC-MALS buffer (10 mM HEPES-KOH pH 7.3, 100 mM KCl, 5 mM $MgCl_2$, 1 mM DTT) with HPLC grade water (Thermofisher). All individual components

(proteins, RNAs) and different combinations of protein:RNA complexes were purified with gel filtration in advance to be injected into SEC-MALS. 240-600 μg of sample in 100 μL volume was injected onto a Superdex 200 10/300 GL gel filtration column (GE healthcare) and at a flow rate 0.5 mL min^{-1}. Multiangle light scattering was detected by a DAWN 8+ detector (Wyatt technology). Data was analyzed with ASTRA (Wyatt technology).

NMR spectroscopy

RNA samples for NMR spectroscopy were lyophilized as sodium salts and dissolved in 420 μL NMR buffer (25 mM HEPES pH 7.3, 150 mM NaCl) and transferred into standard 5mm NMR tubes giving 0.2 to 1 mM sample concentrations. All NMR experiments were recorded on Bruker 600 MHz Avance II+ NMR or Bruker 700 MHz AvanceNeo NMR spectrometers equipped with Prodigy TCI probes. The imino proton resonances in the apo state were assigned by a combination of $^{1}H,^{1}H$- jump and return NOESY experiments (150 ms mixing time, 10°C) and residue-specific $^{15}N^{1}$-guanosine and $^{15}N^{3}$-uridine labeling. The RNA protein complex was prepared by mixing the RNA with one equivalent of ^{15}N labeled $RocC_{14-126}$, followed by size exclusion chromatography. For NMR spectroscopy, the RNA protein complex was concentrated to a volume of 420 μL by ultracentrifugation (molecular weight cut off 3 kDa).

Searching the PDB for examples of protein-RNA interaction motifs observed in RocC-RocR

The duplex portion of the RocR terminator is recognized by an N-capped helix (N-cap motif) in RocC. To search for similar interactions in the PDB, we wrote a Perl script that could identify an N-capping Ser/Thr residue which, together with two C-terminal mainchain NH groups, H-bond with two consecutive phosphate groups (see Ncap_RNA.pl, supplemental files and https://github.com/Glover-Lab/Protein-RNA-interaction-motifs). We searched the entire protein – nucleic acid structure database (all X-ray and cryoEM structures as of April, 2021 – 8935 structures). We visualized the hits in Pymol, aligned on the nucleotides bound by the N-cap. In this way, we uncovered a similar binding interaction between an N-cap motif in the ROQ domain of the T-cell regulatory protein, Roquin, with a region of duplex RNA within a target UTR (Supplementary Figure 8b).

The 3' nucleotide of RocR is recognized by a conserved pocket in RocC that contains a β-hairpin-helix motif composed of amino acids 50-54. To search for similar nucleotide binding

motifs in other structures, we wrote a Perl script that could identify structures that have consecutive backbone NHs that are within hydrogen bonding distance to the O2' and O3' atoms of a nucleotide (see 3pocket.pl, supplemental files and https://github.com/Glover-Lab/Protein-RNA-interaction-motifs). We used this script to scan all protein-RNA and protein-nucleotide structures determined by either X-ray crystallography or cryoEM that were available in the PDB (as of April 2021). We then visualized the hits aligned with the RocC-RocR structure in Pymol and identified an interaction in the eukaryotic ribosomal small subunit as most similar to RocC-RocR. The interaction involves a nucleotide within the 18S rRNA and a β-hairpin-helix motif within the 40S ribosomal protein S24E (Supplementary Figure 8d).

Western blot analysis of RocC and RocC mutants

Cells were grown at 37°C in AYE+Kan and were harvested at an OD_{600} of approximately 1 by centrifugation at 5000 g for 5 min. Pellets were frozen at -80° for 1h and then resuspended in Laemmli 2x (125 mM Tris-HCl pH 6.8; 2% SDS; 1.6% glycerol; 0.01% Bromophenol Blue; 100 mM DTT) to obtain 10^7 cells μL^{-1} and incubated for 10 min at 95°C. Samples (~1.5 x 10^8 cells) were then separated by SDS-polyacrylamide gel electrophoresis (SDS-PAGE). Next, proteins were transferred to a nitrocellulose membrane on a Trans-Blot Turbo Transfer System (Bio-Rad). RocC and RocC mutants were detected with polyclonal anti-RocC antibodies and anti-rabbit IgG [1], HRP conjugated antibodies (Sigma) and revelation was done using the ECL system (Thermo Scientific) according to the manufacturer's instructions. Luminescence signals were acquired using an imaging workstation equipped with a charge-coupled device camera (Thermo Scientific).

REFERENCES

1. Attaiech, L. *et al.* Silencing of natural transformation by an RNA chaperone and a multitarget small RNA. *Proc. Natl. Acad. Sci. U. S. A.* 113, 8813–8818 (2016).
2. Crooks, G., Hon, G., Chandonia, J. & Brenner, S. NCBI GenBank FTP Site\nWebLogo: a sequence logo generator. *Genome Res* 14, 1188–1190 (2004).
3. Juan, P.-A., Attaiech, L. & Charpentier, X. Natural transformation occurs independently of the essential actin-like MreB cytoskeleton in Legionella pneumophila. *Sci. Rep.* 5, 16033 (2015).
4. Bailo, N. *et al.* Scar-Free Genome Editing in Legionella pneumophila. *Methods Mol. Biol.* 1921, 93–105 (2019).
