# Supplementary material for: Structural basis for recognition of transcriptional terminator structures by ProQ/FinO domain RNA chaperones
Source: Nat Commun. 2022 Nov 18;13:7076. doi: 10.1038/s41467-022-34875-5 (PMC9674577; doi:10.1038/s41467-022-34875-5)
Supplement: Supplementary file 3 — Reporting Summary [file 41467_2022_34875_MOESM3_ESM.pdf]

nature portfolio

Corresponding author(s): Mark Glover and Laetitia Attaiech
Last updated by author(s): Nov 4, 2022

Reporting Summary

Nature Portfolio wishes to improve the reproducibility of the work that we publish. This form provides structure for consistency and transparency in reporting. For further information on Nature Portfolio policies, see our Editorial Policies and the Editorial Policy Checklist.

Statistics

For all statistical analyses, confirm that the following items are present in the figure legend, table legend, main text, or Methods section.

n/a	Confirmed	
☐	☒	The exact sample size (*n*) for each experimental group/condition, given as a discrete number and unit of measurement
☐	☒	A statement on whether measurements were taken from distinct samples or whether the same sample was measured repeatedly
☐	☒	The statistical test(s) used AND whether they are one- or two-sided<br>*Only common tests should be described solely by name; describe more complex techniques in the Methods section.*
☒	☐	A description of all covariates tested
☒	☐	A description of any assumptions or corrections, such as tests of normality and adjustment for multiple comparisons
☐	☒	A full description of the statistical parameters including central tendency (e.g. means) or other basic estimates (e.g. regression coefficient) AND variation (e.g. standard deviation) or associated estimates of uncertainty (e.g. confidence intervals)
☐	☒	For null hypothesis testing, the test statistic (e.g. *F*, *t*, *r*) with confidence intervals, effect sizes, degrees of freedom and *P* value noted<br>*Give P values as exact values whenever suitable.*
☒	☐	For Bayesian analysis, information on the choice of priors and Markov chain Monte Carlo settings
☒	☐	For hierarchical and complex designs, identification of the appropriate level for tests and full reporting of outcomes
☒	☐	Estimates of effect sizes (e.g. Cohen's *d*, Pearson's *r*), indicating how they were calculated

Our web collection on statistics for biologists contains articles on many of the points above.

Software and code

Policy information about availability of computer code

Data collection	RocC (24-126) data was collected using HKL3000 (home source). RocC (1-126) data was collected using MxDC (Canadian light source). RocC (14-126) in complex with RNA data was collected using B4 (Advanced light source). FP data were collected using Wallace EnVision manager (Version 1.08 Rev.3)). The intensities of EMSA were measured using Imagequant TL (Version 7.0). ITC data were collected using MicroCal PEAQ-ITC Analysis Software (Ver 1.30.4).
Data analysis	All x-ray crystallographic data were processed using HKL-2000 (version 720) and Phenix suite (Version 1.20.1-4487). Determined structure was analyzed by Pymol (Version 2.5.2) and Coot (Version 0.9). The script used for PDB searching was deposited on Github (https://github.com/Glover-Lab/Protein-RNA-interaction-motifs). FP data were analyzed by following website: https://www.aatbio.com/tools/four-parameter-logistic-4pl-curve-regression-online-calculator. EMSA data were analyzed using GraphPad Prism (Version 5.00). ITC data were analyzed using MicroCal PEAQ-ITC Analysis Software (Ver 1.30.4).

For manuscripts utilizing custom algorithms or software that are central to the research but not yet described in published literature, software must be made available to editors and reviewers. We strongly encourage code deposition in a community repository (e.g. GitHub). See the Nature Portfolio guidelines for submitting code & software for further information.

March 2021

Data

Policy information about availability of data

All manuscripts must include a data availability statement. This statement should provide the following information, where applicable:

- Accession codes, unique identifiers, or web links for publicly available datasets
- A description of any restrictions on data availability
- For clinical datasets or third party data, please ensure that the statement adheres to our policy

The three atomic structures presented in this publication were deposited in the Protein Data Bank : RocC(24-126) - PDB accession code: 7RGS, RocC(1-126) - PDB accession code: 7RGT, and RocC(14-126)/RocR(9bp-tet) - PDB accession code: 7RGU. All data are available upon request.

Field-specific reporting

Please select the one below that is the best fit for your research. If you are not sure, read the appropriate sections before making your selection.

☒ Life sciences ☐ Behavioural & social sciences ☐ Ecological, evolutionary & environmental sciences

For a reference copy of the document with all sections, see nature.com/documents/nr-reporting-summary-flat.pdf

Life sciences study design

All studies must disclose on these points even when the disclosure is negative.

Sample size	Sufficient sample sizes for binding assays were used to create sigmoidal binding curves. The sample sizes for binding assays were referred by the published study (https://doi.org/10.1073/pnas.160162611). The final sample sizes were determined based on experiments.
Data exclusions	No data were excluded.
Replication	All in vitro binding assays were replicated by three independent experiments, as we indicated in the corresponding figure legends. All transformation assays were repeated at list twice on 2 independent clones and/or with 2 types of donor DNA. X-ray crystallographic structure was determined by standard procedures and does not require replicates. All binding samples for binding experiments for statistics were replicated at least three times.
Randomization	Randomization is not relevant to our in vitro binding studies, in vivo functional assay, and structural study.
Blinding	Blinding is not relevant to our in vitro binding studies, in vivo functional assay, and structural study.

Reporting for specific materials, systems and methods

We require information from authors about some types of materials, experimental systems and methods used in many studies. Here, indicate whether each material, system or method listed is relevant to your study. If you are not sure if a list item applies to your research, read the appropriate section before selecting a response.

Materials & experimental systems

n/a	Involved in the study	
☐	☒	Antibodies
☒	☐	Eukaryotic cell lines
☒	☐	Palaeontology and archaeology
☒	☐	Animals and other organisms
☒	☐	Human research participants
☒	☐	Clinical data
☒	☐	Dual use research of concern

Methods

n/a	Involved in the study	
☒	☐	ChIP-seq
☒	☐	Flow cytometry
☒	☐	MRI-based neuroimaging

Antibodies

Antibodies used	Anti-RocC antibodies
Validation	They are polyclonal antibodies from rabbit, generated against a purified His-tagged RocC protein. The method for their obtention and results of their validation is described in Attaiech et al., 2016 (DOI: 10.1073/pnas.1601626113).
